# The utilization of 3D-printed arthrodesis prostheses in the repair and reconstruction of malignant tumors in proximal humerus

**DOI:** 10.3389/fonc.2025.1596530

**Published:** 2025-06-18

**Authors:** Guolong Bin, Bin Liu, Zhenchao Yuan, Jiachang Tan

**Affiliations:** Department of Bone and Soft Tissue Surgery, Guangxi Medical University Cancer Hospital, Nanning, China

**Keywords:** bone tumor, arthrodesis, shoulder, 3D printing, prostheses

## Abstract

**Background:**

The utilization of 3D-printed arthrodesis prostheses represent a viable reconstructive option for patients who are unable to retain the deltoid muscle or axillary nerve following the resection of malignant tumors in the proximal humerus. However, there exists a limited body of literature regarding the application of this prosthetic type. In this study, we assessed the effectiveness of 3D-printed arthrodesis prostheses in the repair and reconstruction of the proximal humerus post-tumor resection. Additionally, we investigated the incidence of postoperative complications and the enhancements associated with the prostheses.

**Patients and methods:**

This study retrospectively examined a cohort of 12 patients diagnosed with malignant tumors of the proximal humerus, who underwent reconstruction utilizing 3D-printed arthrodesis prostheses at our institution between January 2020 and December 2022. The design of the prostheses were conducted preoperatively through computer-aided design (CAD) technology, among other methods. Each patient underwent intraoperative resection of the tumor-affected segment followed by reconstruction with the 3D-printed arthrodesis prostheses. Postoperative follow-up assessments were conducted to evaluate the range of motion (ROM) of the shoulder joint, assess shoulder joint functionality, document the occurrence of complications, and analyze potential risk factors associated with these outcomes.

**Result:**

All 12 patients underwent the procedure without complications, with an average operation duration of (172.5 ± 58.9) min (range 130-240 min). The average blood loss recorded was (450.3 ± 118.4) ml (range 200-1000 ml), and the average length of the osteotomy at the proximal humerus was (16.5 ± 1.2) cm (range 15-19 cm). The follow-up period for all patients averaged (26.2 ± 14.5) months (range 12-49 months). At the final follow-up, the average active shoulder joint abduction among the twelve patients was 75.2° ± 12.3°(range 45°-110°), while the average anterior flexion was 70.8° ± 18.4°(range 60°-125°). The average American Shoulder and Elbow Surgeons (ASES) score was 75.2 ± 6.3 points (range 63-90 points), and the Musculoskeletal Tumor Society (MSTS-93) score averaged 22.1 ± 2.5points (range 21-27 points). According to the complication classification system for post-prosthetic reconstruction proposed by Henderson, the group exhibited one case of type I, two cases of type II, two cases of type III, and two cases of type IV complications.

**Conclusion:**

The utilization of 3D-printed arthrodesis prostheses in the repair and reconstruction of malignant tumors in proximal humerus has the potential to enhance postoperative functionality. However, the prevalence of complications associated with these prostheses remains significant. Therefore, there is a pressing need for advancements in both prostheses design and surgical protocols to optimize clinical outcomes.

## Introduction

The proximal humerus constitutes the most prevalent primary site for neoplastic lesions within the shoulder girdle region and ranks as the third most frequent anatomical localization for osteosarcoma among skeletal malignancies (1).

In the management of proximal humeral malignancies, the deltoid muscle and axillary nerve are frequently compromised by tumor infiltration, necessitating extensive resection of adjacent soft tissues, including the deltoid, to achieve histologically negative surgical margins. Conventional hemiarthroplasty demonstrates suboptimal postoperative functional outcomes, characterized by compromised glenohumeral stability and a high incidence of complications such as prosthetic migration and dislocation (2). As an alternative surgical strategy, glenohumeral arthrodesis has been proposed to restore mechanical stability, mitigate pain, and preserve partial upper extremity functionality through rigid osseous fixation (3–5, 19).

Glenohumeral arthrodesis may utilize autografts or allografts for biological reconstruction; however, this approach has been associated with postoperative complications such as fracture and nonunion (5). Furthermore, the clinical application of autologous fibular grafts is constrained by limited donor availability, particularly in cases involving extensive bone defects exceeding 13 cm in length, where such grafts are anatomically and functionally inadequate (6). Additionally, the newly formed osseous tissue frequently exhibits insufficient mechanical strength to withstand physiological loads, compromising long-term structural stability.

To address these limitations, Guo et al. implemented 3D-printed arthrodesis prostheses for glenohumeral fusion, demonstrating favorable early postoperative functional outcomes with no evidence of prosthetic loosening, fracture, or infection during follow-up (7). To further evaluate the therapeutic efficacy of this prosthetic replacement protocol, a retrospective analysis was conducted on 12 patients undergoing oncological reconstruction with 3D-printed arthrodesis prostheses at our institution. The study objectives were twofold: (1) to assess the short- to medium-term functional recovery and reconstructive outcomes following proximal humeral malignancy resection with 3D-printed arthrodesis prostheses; (2) to elucidate the design principles and biomechanical advantages inherent to 3D-printed arthrodesis prostheses in arthrodesis applications.

## Patients and methods

A cohort of twenty patients diagnosed with malignant tumors in proximal humerus underwent reconstruction using 3D-printed arthrodesis prostheses at the Department of Bone and Soft Tissue Surgery, Guangxi Medical University Cancer Hospital, between January 2020 and December 2022. The selection of patients was based on specific inclusion and exclusion criteria. The inclusion criteria comprised: 1. patients diagnosed with primary proximal humeral malignancies; 2. cases where preservation of the deltoid muscle or axillary nerve was not feasible; 3.availability of complete imaging and follow-up data. Conversely, the exclusion criteria included: 1. patients in whom the deltoid muscle, axillary nerve, and a majority of the surrounding tissues of the scapular girdle could be preserved during tumor resection; 2. those with extensive metastatic disease, poor systemic health, or an inability to tolerate surgical intervention; 3. cases involving amputation or invasion of the distal humerus; 4. Secondary, metastatic, or multifocal malignant neoplasms. Ultimately, twelve patients were included in the study, comprising eight males and four females, with a mean age of (27.7 ± 14.3) years (range 14-55 years). All patients presented with unilateral disease, with nine cases on the left side and three on the right. Each patient received a preoperative diagnosis of osteosarcoma confirmed through puncture pathology. Prior to surgery, all patients underwent standardized neoadjuvant chemotherapy, during which one patient experienced a pathological fracture that was temporarily stabilized with a splint until the preoperative phase. The demographic and clinical characteristics of the patients are summarized in **Table 1**. The research received approval from the Ethics Committee of Guangxi Medical University Cancer Hospital, and all participants provided informed consent (Ethics No.2025360).

**Table 1:** Fundamental patient information.

Variables	Values
Gender [N (%)]	
Male	8 (67.0)
Female	4 (33.0)
Age (yr, mean ± SD)	27.7 ± 14.3
Follow-up time (month, mean ± SD)	26.2 ± 14.5
Histological diagnosis [N (%)]	
Osteosarcoma	12 (100.0)
Pathological fracture [N(%)]	1 (8.3)
Greatest axial diameter of the tumor (mm, mean ± SD)	60.5 ± 31.6
Operative duration (min, mean ± SD)	172.5 ± 58.9
Intraoperative hemorrhage (ml, mean ± SD)	450.3 ± 118.4
Preservation of axillary nerve [N(%)]	0 (0.0)
Proportion of resection (%, mean ± SD)	42.4 ± 8.3

### Design of the arthrodesis prostheses

All 3D-printed arthrodesis prostheses were collaboratively designed by the Department of Bone and Soft Tissue Surgery at Guangxi Medical University Cancer Hospital and Beijing Chunli Zhengda Medical Instrument Co., Ltd. These prostheses were fabricated using standardized modular components, with no individualized customization implemented in the manufacturing process.

**Figure 1:**
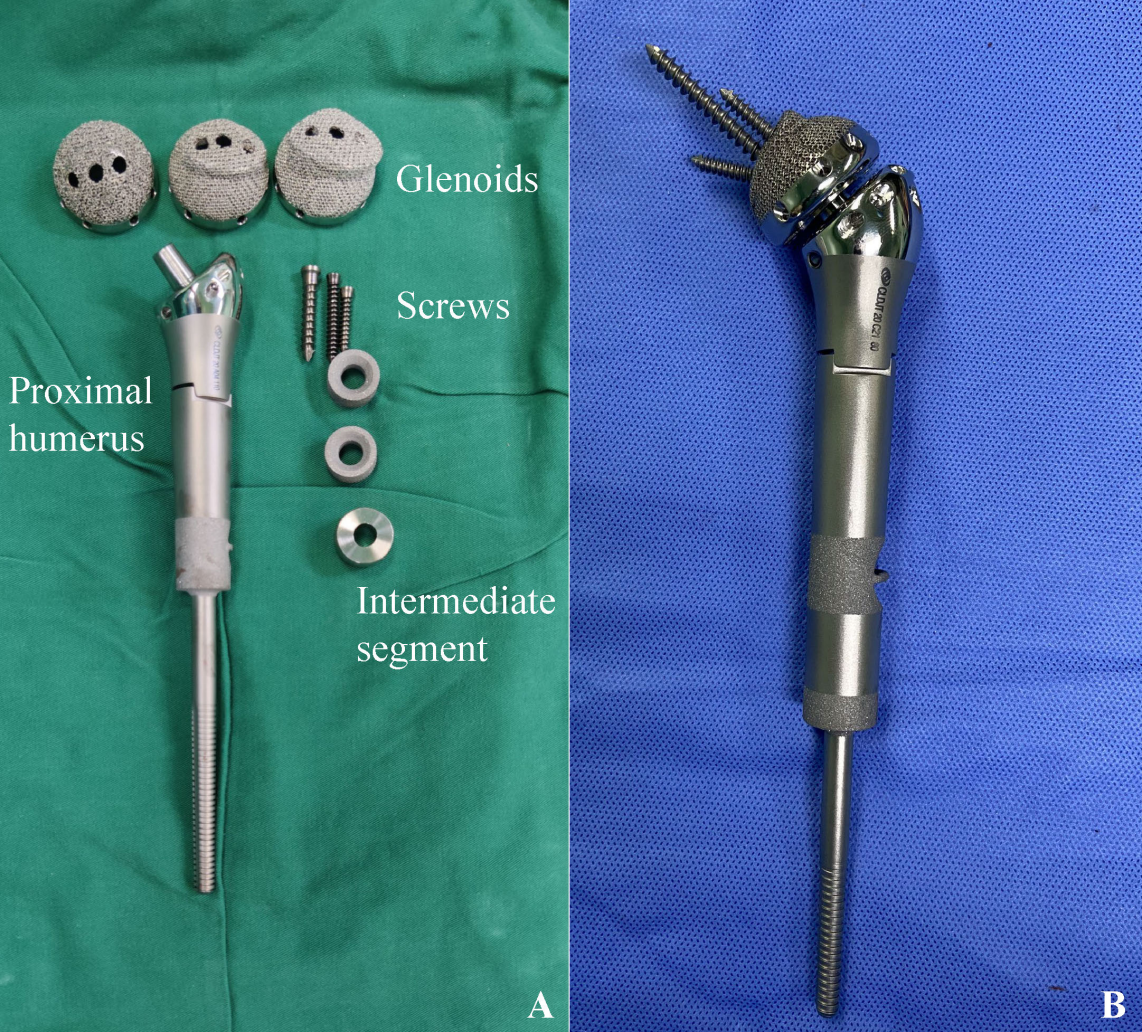
3D-printed arthrodesis prosthesis. **(A)** Glenoid component,screws,intermediate segment and humeral component. The intermediate segment serves as a connector between the glenoid and humeral components through the utilization of a Morse taper; **(B)** The constructed arthrodesis prosthesis.

3D-printed arthrodesis prostheses consist of glenoid components, intermediate segments and humeral components (**Figure 1**). The humeral component utilized in this study was a conventional hemiarthroplasty cemented design, featuring a distal bone contact area that was coated. Additionally, two 3D-printed rings, ranging in size from 0.5 to 2 cm, were fabricated preoperatively. These rings were designed to be nested within the upper section of the prostheses stem, allowing for precise adjustments to the length of the prostheses. The glenoid component is fabricated from Ti6Al4V utilizing electron beam melting (EBM) technology and is designed in a modular format comprising three sequential sizes. The configuration of the external interface was specifically engineered to correspond with the morphology of the articular surface of the scapular glenoid. It featured an appropriate pore size of 500 μm and a porosity rate of 60%, which promoted the ingrowth of bone. The medial aspect of the glenoid component incorporates a Morse taper design, with three screw fixation holes positioned within the scapular body to achieve rigid osseous stabilization. The intermediate segment consisted of a metallic plug featuring a Morse taper, which served to link the glenoid component with the humeral component. The three components were joined through the impaction of the Morse tapers.

### Surgical procedures

Following the administration of general anesthesia, the patient was positioned in a semi-recumbent posture, and the surgical site was disinfected and draped appropriately. An S-shaped incision was executed on the skin over the upper portion of the humerus, encompassing the original puncture biopsy site. The incision proceeded through the skin, subcutaneous tissue, and deep fascia in a sequential manner. Based on the resection parameters established from preoperative imaging, the tumor was meticulously dissected from the surrounding healthy tissues, adhering to the principle of achieving tumor-free margins. Careful attention was given to the mobilization and preservation of uninvolved nerves during the excision of the tumor, along with the affected deltoid muscle or axillary nerves. The surgical procedure aimed to fully expose the margins of the glenoid, with the articular cartilage being meticulously abraded until punctate bleeding from the subchondral bone was achieved. The glenoid prosthesis is positioned with careful consideration of the center of rotation and the coating of the prosthesis to ensure optimal contact, while also adjusting the posterior tilt angle to appropriate specifications. The fixation of the glenoid prosthesis is achieved by initially placing the intermediate screw, followed by the superior and inferior screws. The residual humeral structure is fully expanded and secured using bone cement. Following the implantation of a humeral prosthesis of suitable length, a comprehensive assessment of joint stability is conducted through movement in all directions. Subsequently, a synthetic mesh is applied around the prosthesis, and the surrounding musculature, along with the rotator cuff stump, is sutured to both the periphery of the prosthesis and the mesh to enhance joint stability and eliminate any potential dead space. The surgical field is thoroughly irrigated, drains are inserted, and the various layers of tissue are meticulously sutured in succession.

### Postoperative management and follow−up

The drainage system was extracted once the drainage output fell below 50 ml within a 24-hour period. The patient was fitted with an abduction brace for a duration of eight weeks, during which active mobilization and strength training exercises for the elbow and hand were commenced. Following the removal of the brace, a sling was applied, and both active and passive mobilization of the shoulder was initiated. Patients typically undergo follow-up evaluations every three months during the initial two years, followed by assessments every four months in the third year, every six months in the fourth year, and annually thereafter.

### Assessment methodology

All patients consistently attended the hospital for postoperative evaluations, which included X-ray and CT imaging assessments. These evaluations aimed to determine the presence of any local tumor recurrence, distant metastasis, as well as to assess the integrity of the prostheses in terms of loosening, fracture, and bone healing. Additionally, we employed the MSTS-93 score and the ASES score to assess the patients’ shoulder functionality and to measure the range of motion (ROM) of the shoulder joint. The measurements, including shoulder abduction angle, shoulder anterior flexion angle, MSTS-93 score, and ASES score, conformed to a normal distribution and were presented as mean ± standard deviation ( $\bar{x}$ ± s).

Range of motion (ROM) measurements were conducted by a single senior orthopedic surgeon utilizing a universal goniometer, in strict adherence to the standardized protocols outlined by the American Academy of Orthopaedic Surgeons. Subdomains of the ASES and MSTS-93 scoring questionnaires underwent systematic refinement and were independently evaluated by a uniformly trained research team, which had completed rigorous standardization training in the administration of these assessment tools prior to study initiation.

## Result

### Operational details

The surgical procedure was conducted without complications, with an average operation duration of (172.5 ± 58.9) min (range: 130-240 min). The mean blood loss recorded was (450.3 ± 118.4) ml (range: 200-1000 ml). Additionally, the average length of the osteotomy was measured at (16.5 ± 1.2) cm (range: 15-19 cm).

### Oncological survival and complication

The average follow-up period for the patients was (26.2 ± 14.5) months (range: 12-49 months). At the last follow-up, four patients (cases 1, 2, 4, 5) exhibited postoperative lung metastasis. Among these, one patient (case 1) was treated with oral Anilotinib and is currently alive with tumor. Three patients (cases 2, 4, 5) succumbed to their condition at 12, 17, and 19 months postoperatively, respectively. Utilizing the classification of postoperative complications established by Henderson et al (9), the complications observed in this cohort were categorized as follows: 1 case of type I, 2 cases of type II, 2 cases of type III, and 2 cases of type IV (**Table 2**).

**Table 2:** Patients and associated postoperative complications.

No.	Soft-tissue failure (Type I)	Aseptic loosening (Type II)	Structural failure (Type III)	Infection (Type IV)	Tumor progression (Type V)	Revision
1			Taper detachment	Periprosthetic Joint Infection		Open Reduction/Partial Prosthesis Removal
2	Implant Exposure		Taper detachment			Open Reduction/Latissimus Dorsi Flap
3		Distal Aseptic Loosening				
4		Distal Aseptic Loosening				
5				Periprosthetic Joint Infection		Partial Prosthesis Removal
Total	1	2	2	2		

### Functional outcomes

At the last follow-up assessment, the mean active abduction angle of the shoulder joint was 75.2° ± 12.3° (range 45°-110°). The mean active forward flexion was 70.8° ± 18.4° (range 60°-125°). The average ASES score was 75.2 ± 6.3 (range 63-90). Additionally, the mean MSTS-93 score was 22.1 ± 2.5 (range 21-27).

## Discussion

Historically, proximal humeral malignancies involving the deltoid muscle and axillary nerve were managed with hemiarthroplasty or bone cement spacer reconstruction. However, owing to extensive soft tissue deficits in the shoulder region, these conventional approaches often resulted in markedly compromised postoperative functional outcomes, including restricted glenohumeral mobility and diminished stability (2). To address these limitations, the authors implemented 3D-printed arthrodesis prostheses as a reconstructive strategy, aiming to optimize biomechanical integrity and restore functional capacity following tumor resection.

**Figure 2:**
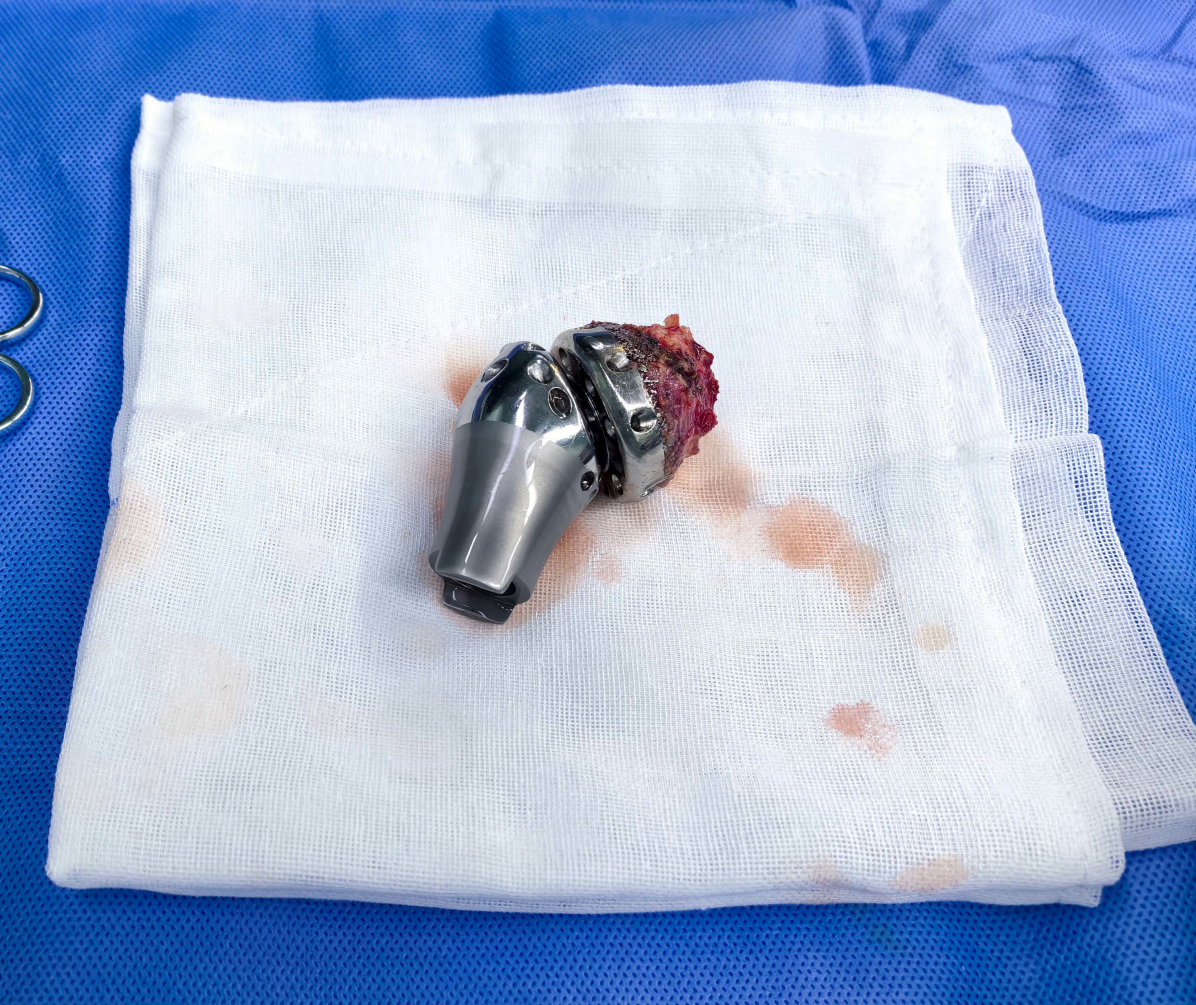
The excised specimens exhibited notable osseous proliferation.

**Figure 3:**
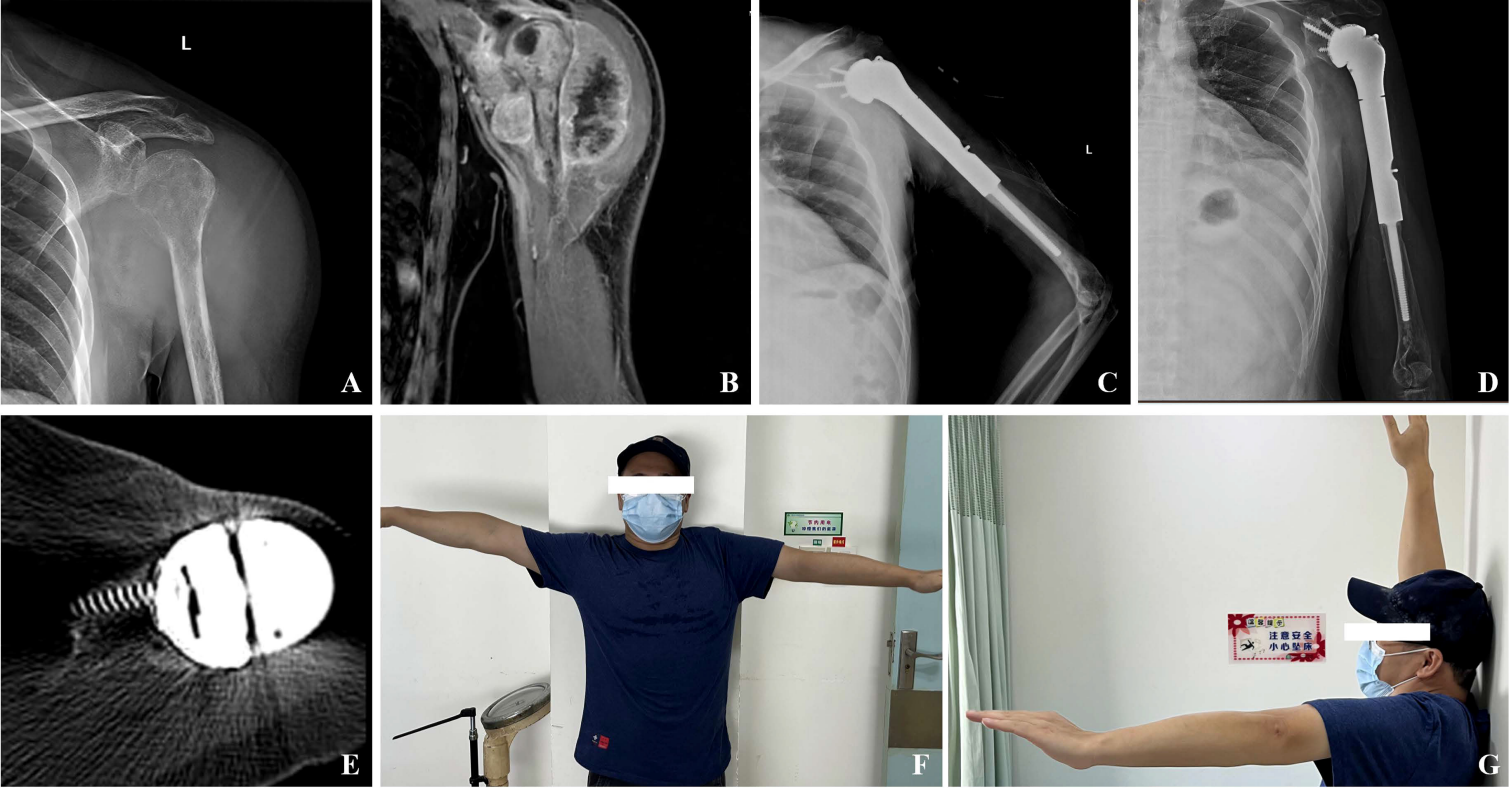
**(A)** Preoperative X-ray showing bone destruction of the left proximal humerus; **(B)** MRI suggests extensive tumor invasion with deltoid involvement; **(C)** A follow-up X-ray conducted one week post-surgery indicated that the prosthesis was securely positioned; **(D)** Radiographic evaluations conducted 36 months following the surgical procedure indicated that the prosthesis was appropriately positioned, exhibiting no signs of loosening or displacement; **(E)** CT scan 36 months after surgery showed new bone formation around the porous interface of the glenoid component; **(F, G)** Maximum shoulder abduction and forward flexion of up to 90°can be observed at the last follow-up.

The shoulder prostheses comprise three components: the glenoid components, intermediate segments, and humeral prostheses. The application of 3D printing technology facilitates the fabrication of glenoid components with highly porous architectures, which promote osteointegration through enhanced bone ingrowth. This structural design, combined with surface coating modifications, significantly improves the bone-implant integration rate at the glenoid-scapular interface and augments early proximal stability. Postoperative imaging evaluations at the final follow-up revealed no instances of glenoid component loosening in all 12 patients. Radiographic evidence demonstrated robust osseointegration at the glenoid-scapular interface, characterized by peri-prosthetic bone formation within the porous scaffold, achieving effective fusion between the autologous bone and the prostheses. In cases requiring debridement and glenoid component explantation due to periprosthetic infection, histological analysis of retrieved specimens confirmed intimate bone-implant integration, necessitating partial osteotomy for component removal. Macroscopic examination further corroborated bone ingrowth within the porous matrix (**Figure 2**). The biomechanical rationale for glenohumeral arthrodesis lies in substituting scapulothoracic articulation for the native glenohumeral joint, thereby preserving partial abduction and forward flexion through compensatory motion of the scapulothoracic complex (2). A stable glenoid-scapular interface achieved via rigid osseointegration contributes to enhanced shoulder stability and functional range of motion. Comparative studies by Cannon et al. involving 83 hemiarthroplasty cases reported postoperative mean abduction and forward flexion angles of 41°and 42°, respectively (8). Similarly, Stevenson et al. documented mean abduction of 57°and forward flexion of 63°in 9 patients undergoing fibular autograft arthrodesis (10). In contrast, the current cohort demonstrated superior early functional outcomes, with mean postoperative abduction of 75.2° ± 12.3°and forward flexion of 70.8° ± 18.4°. Quantitative assessments using the MSTS-93 scoring system (22.1 ± 2.5) and the ASES score (75.2 ± 6.3) confirmed significant improvements in shoulder functionality and mobility (**Figure 3**).

The shoulder prostheses deliver immediate postoperative joint stability and mechanical integrity; however, the inherent characteristics of the Morse taper design elevate risks of taper detachment. Guo et al. postulate that taper detachment may arise from inadequate torsional resistance at the Morse taper interface. Intraoperative validation of taper stability and circumferential reinforcement of the glenoid component with LARS ligaments can reduce dissociation risks. Furthermore, modified 3D-printed arthrodesis prostheses, incorporating a long transfixing screw inserted laterally through the humeral head to rigidly anchor the glenoid-humeral assembly to the scapula, have been advocated to enhance proximal fixation and mitigate Morse taper detachment (7). Wang et al. observed no proximal complications in 16 arthrodesis cases utilizing adjunctive proximal plate fixation at final follow-up (14). In this cohort, taper detachment occurred in two early-stage cases, likely attributable to suboptimal glenoid implantation, which generated excessive shear forces and localized stress concentration at the Morse taper junction. Consensus across studies underscores that precise intraoperative alignment of the fusion site is pivotal to clinical outcomes and complication avoidance (11–13). Anatomically accurate positioning may optimize Morse taper engagement, thereby reducing stress transmission during functional loading. Crucially, malpositioned glenoid components risk progressive stress accumulation on auxiliary fixation screws, even with long transfixing screws, predisposing to late screw fatigue or loosening. Preoperative virtual simulation of implant placement, combined with patient-specific prostheses designs guided by anatomical landmarks or computer-assisted navigation, may improve positional accuracy and minimize detachment-related complications.

Extensive resection of periscapular soft tissues frequently results in critical volumetric deficits, leading to compromised vascularity and diminished infection resistance at the surgical site. These conditions elevate risks of impaired wound healing and prostheses-related complications, including instability and dislocation due to insufficient soft tissue encapsulation (14). In this series, one patient (Case 1) developed periprosthetic infection following multiple revisions for recurrent taper detachment, while another (Case 5) exhibited periprosthetic infection six months postoperatively. After repeated unsuccessful debridements, the glenoid component and partial humeral stem were explanted, with antibiotic-impregnated cement applied to the residual prosthesis to achieve infection control. A third patient (Case 2) presented with prosthesis exposure, which was successfully managed via pedicled latissimus dorsi myocutaneous flap coverage, restoring regional perfusion without subsequent complications. Pedicled latissimus dorsi flaps provide robust vascularized tissue coverage, effectively mitigating risks of prostheses exposure secondary to cutaneous atrophy or extensive tissue loss. Wang et al. demonstrated that latissimus dorsi flap integration during arthrodesis significantly reduces infection rates and enhances postoperative shoulder function through partial compensation for deltoid deficits (14). Similarly, Behnam et al. reported zero complications in six patients undergoing prosthetic reconstruction with concomitant primary latissimus dorsi flap reconstruction (16). These findings support our recommendation for combining 3D-printed arthrodesis prostheses with primary latissimus dorsi flap coverage to minimize exposure risks, prevent deep infections, optimize prosthetic stability, and partially restore shoulder biomechanical function.

**Figure 4:**
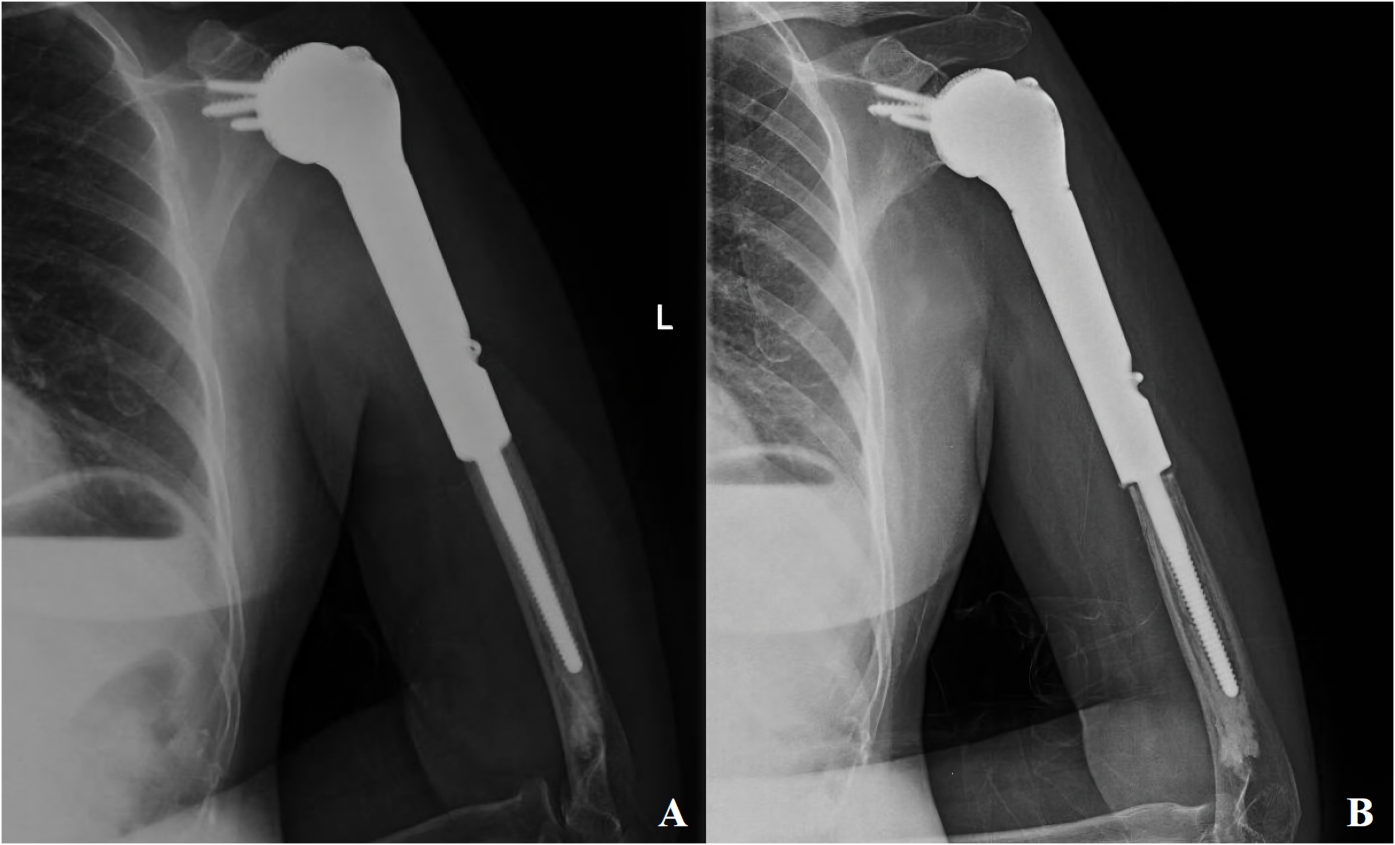
Patient, female, 55 years old, diagnosed with osteosarcoma of the proximal left humerus **(A)** postoperative follow-up x-ray; **(B)** aseptic loosening of the distal end of the prosthesis as seen on a second follow-up x-ray at 8 months postoperatively.

Follow-up radiographic evaluation in this cohort revealed progressive periprosthetic lucency around the distal humeral stems of two patients, though neither exhibited symptomatic pain (**Figure 4**). Current evidence suggests that multifocal or progressive radiolucent lines adjacent to prosthetic stems may indicate impending aseptic loosening (17, 18). Blewitt et al. postulated that excessive functional loading of arthrodesis constructs could induce elevated stress concentrations in distal stems, predisposing to fatigue fractures (20). Lin et al. demonstrated through axial loading simulations of novel 3D-printed arthrodesis prostheses that stress gradients increase progressively from proximal to distal regions, with pronounced stress concentration at the distal stem interface (15). In this series, rigid osseointegration at the glenoid-scapular interface redirected functional stresses toward the distal stem, potentially exacerbating stress shielding effects and contributing to distal loosening. Pareatumbee et al. theorized that extended osteotomy lengths and prolonged stem dimensions reduce mechanical stimuli to residual bone, accelerating stress shielding-induced bone resorption and prosthetic failure (21). Notably, the two affected cases exhibited osteotomy lengths of 17 cm and 19 cm, respectively, with extensive stem implantation likely intensifying distal stress shielding. Montemaggi et al. reported reduced stress shielding in 46 patients using shorter stems combined with intramedullary autograft augmentation, observing only three cases of stress shielding with one mild bone resorption (22). Given the mean osteotomy length of 16.5 ± 1.2 cm and limited residual humeral fixation length in this cohort, distal loosening risks remain clinically relevant. To mitigate these risks, cementless stems or allograft-augmented interface reconstruction could be considered for distal humeral fixation. While supplemental extramedullary plating may reduce early stress shielding in cases with limited fixation length, such constructs risk stress concentration at plate-stem junctions, potentially increasing periprosthetic fracture rates.

This study has several limitations. First, the limited cohort size may compromise statistical power and restrict the generalizability of conclusions, thereby precluding comprehensive statistical analyses. Subsequent investigations should employ multicenter collaborations to expand sample sizes for robust validation. Second, the relatively short follow-up duration necessitates extended surveillance to evaluate delayed complications, long-term functional outcomes, and the durability of 3D-printed arthrodesis prostheses in proximal humeral oncological reconstruction. Longitudinal studies are imperative to elucidate optimal fixation strategies and late-term efficacy of this reconstructive approach.

## Conclusion

The utilization of 3D-printed arthrodesis prostheses in the repair and reconstruction of malignant tumors in proximal humerus has the potential to enhance postoperative functionality. However, the prevalence of complications associated with these prostheses remains significant. Therefore, there is a pressing need for advancements in both prostheses design and surgical protocols to optimize clinical outcomes.

## Data Availability

The original contributions presented in the study are included in the article/supplementary material. Further inquiries can be directed to the corresponding authors.
